# Cognitive reserve and *TMEM106B* genotype modulate brain damage in presymptomatic frontotemporal dementia: a GENFI study

**DOI:** 10.1093/brain/awx103

**Published:** 2017-04-27

**Authors:** Enrico Premi, Mario Grassi, John van Swieten, Daniela Galimberti, Caroline Graff, Mario Masellis, Carmela Tartaglia, Fabrizio Tagliavini, James B. Rowe, Robert Laforce Jr, Elizabeth Finger, Giovanni B. Frisoni, Alexandre de Mendonça, Sandro Sorbi, Stefano Gazzina, Maura Cosseddu, Silvana Archetti, Roberto Gasparotti, Marta Manes, Antonella Alberici, Manuel J. Cardoso, Martina Bocchetta, David M. Cash, Sebastian Ourselin, Alessandro Padovani, Jonathan D. Rohrer, Barbara Borroni, on behalf of the Genetic FTD Initiative (GENFI)

**Affiliations:** 1 Neurology Unit, Department of Clinical and Experimental Sciences, University of Brescia, Brescia, Italy; 2 Department of Brain and Behavioral Science, Medical and Genomic Statistics Unit, University of Pavia, Italy; 3 Department of Neurology, Erasmus Medical Center, Rotterdam, The Netherlands; 4 Department of Pathophysiology and Transplantation, “Dino Ferrari” Center, University of Milan, Fondazione Cà Granda, IRCCS Ospedale Maggiore Policlinico, Milan, Italy; 5 Karolinska Institutet, Department NVS, Center for Alzheimer Research, Division of Neurogenetics, Sweden; 6 Department of Geriatric Medicine, Karolinska University Hospital-Huddinge, Stockholm, Sweden; 7 LC Campbell Cognitive Neurology Research Unit, Sunnybrook Research Institute, Toronto, ON, Canada; 8 Toronto Western Hospital, Tanz Centre for Research in Neurodegenerative Disease, Toronto, ON, Canada; 9 Fondazione Istituto di Ricovero e Cura a Carattere Scientifico Istituto Neurologico Carlo Besta, Milano, Italy; 10 Department of Clinical Neurosciences, University of Cambridge, Cambridge, UK; 11 Clinique Interdisciplinaire de Mémoire, Département des Sciences Neurologiques, CHU de Québec, and Faculté de Médecine, Université Laval, QC, Canada; 12 Department of Clinical Neurological Sciences, University of Western Ontario, London, ON, Canada; 13 Istituto di Ricovero e Cura a Carattere Scientifico (IRCCS) Istituto Centro San Giovanni di Dio Fatebenefratelli, Brescia, Italy; 14 Memory Clinic and LANVIE-Laboratory of Neuroimaging of Aging, University Hospitals and University of Geneva, Geneva, Switzerland; 15 Faculty of Medicine, University of Lisbon, Lisbon, Portugal; 16 Department of Neuroscience, Psychology, Drug Research and Child Health, University of Florence, Florence, Italy; 17 Istituto di Ricovero e Cura a Carattere Scientifico (IRCCS) “Don Gnocchi”, Florence, Italy; 18 Department of Laboratories, III Laboratory of Analysis, Brescia Hospital, Brescia, Italy; 19 Neuroradiology Unit, University of Brescia, Italy; 20 Dementia Research Centre, UCL Institute of Neurology, London, UK; 21 Translational Imaging Group, Centre for Medical Image Computing, Department of Computer Science, UCL, London, UK

**Keywords:** frontotemporal dementia, genetics, cognitive reserve, TMEM106b, structural MRI

## Abstract

Frontotemporal dementia is a heterogeneous neurodegenerative disorder with around a third of cases having autosomal dominant inheritance. There is wide variability in phenotype even within affected families, raising questions about the determinants of the progression of disease and age at onset. It has been recently demonstrated that cognitive reserve, as measured by years of formal schooling, can counteract the ongoing pathological process. The *TMEM106B* genotype has also been found to be a modifier of the age at disease onset in frontotemporal dementia patients with TDP-43 pathology. This study therefore aimed to elucidate the modulating effect of environment (i.e. cognitive reserve as measured by educational attainment) and genetic background (i.e. *TMEM106B* polymorphism, rs1990622 T/C) on grey matter volume in a large cohort of presymptomatic subjects bearing frontotemporal dementia-related pathogenic mutations. Two hundred and thirty-one participants from the GENFI study were included: 108 presymptomatic *MAPT*, *GRN*, and *C9orf72* mutation carriers and 123 non-carriers. For each subject, cortical and subcortical grey matter volumes were generated using a parcellation of the volumetric T_1_-weighted magnetic resonance imaging brain scan. *TMEM106B* genotyping was carried out, and years of education recorded. First, we obtained a composite measure of grey matter volume by graph-Laplacian principal component analysis, and then fitted a linear mixed-effect interaction model, considering the role of (i) genetic status; (ii) educational attainment; and (iii) *TMEM106B* genotype on grey matter volume. The presence of a mutation was associated with a lower grey matter volume (*P = *0.002), even in presymptomatic subjects. Education directly affected grey matter volume in all the samples (*P = *0.02) with lower education attainment being associated with lower volumes. *TMEM106B* genotype did not influence grey matter volume directly on its own but in mutation carriers it modulated the slope of the correlation between education and grey matter volume (*P = *0.007). Together, these results indicate that brain atrophy in presymptomatic carriers of common frontotemporal dementia mutations is affected by both genetic and environmental factors such that *TMEM106B* enhances the benefit of cognitive reserve on brain structure. These findings should be considered in evaluating outcomes in future disease-modifying trials, and support the search for protective mechanisms in people at risk of dementia that might facilitate new therapeutic strategies.

## Teaser

Frontotemporal dementia (FTD) shows substantial phenotypic variability. In a multicentre study, Premi *et al*. explore the effect of cognitive reserve and *TMEM106B* genotype in modulating grey matter volume in presymptomatic FTD. Environmental as well as genetic factors affect rates of brain atrophy, suggesting a possible strategy for delaying disease onset.

## Introduction

Frontotemporal dementia (FTD) is a neurodegenerative disorder characterized by neuronal loss in the frontal and temporal lobes (Hodges *et al.*, 2004; Rohrer *et al.*, 2011; Warren *et al.*, 2013). It presents clinically with behavioural symptoms, deficits of executive functions and language impairment, and in some cases, with motor neuron disease, progressive supranuclear palsy or corticobasal syndrome (Seelaar *et al.*, 2011). Up to 40% of cases have a family history of dementia, with an autosomal dominant inheritance in around a third of patients (Stevens *et al.*, 1998). Mutations within microtubule-associated protein tau (*MAPT*) (Hutton *et al.*, 1998), granulin (*GRN*) (Baker *et al.*, 2006; Cruts *et al.*, 2006), and chromosome 9 open reading frame 72 (*C9orf72*) (DeJesus-Hernandez *et al.*, 2011; Renton *et al.*, 2011) are proven major causes of genetic FTD, accounting for 10–20% of all FTD cases. *MAPT* mutations lead to FTD with neuronal tau inclusions, while *GRN* and *C9orf72* are associated with intraneuronal TAR DNA-binding protein 43 (TDP-43) inclusions (Baborie *et al.*, 2011).

Recently, it has been demonstrated in the Genetic Frontotemporal Dementia Initiative (GENFI) study that grey matter and cognitive changes can be identified 5–10 years before the expected onset of symptoms in adults at risk of genetic FTD (Rohrer *et al.*, 2015), and even earlier for those with *C9orf72* expansions. However, there is wide variation in the age at onset within families, and possible modifiers of disease progression (including genetic and environmental factors) have yet to be investigated. Such modifiers will be important for several reasons: to properly define biomarkers that can stage presymptomatic disease and track disease progression, to correctly identify individuals most suitable for clinical trials, and to reduce heterogeneity and increase the statistical power of analyses of such trials.

Cognitive reserve and genetic factors have both been proposed as moderators of the onset of disease. Cognitive reserve is a theoretical concept proposing that certain lifetime experiences, including education, individual intelligence quotient, degree of literacy, and occupational attainment, increase the flexibility, efficiency, and capacity of brain networks, thereby allowing individuals with higher cognitive reserve to sustain greater levels of brain pathology before showing clinical impairment (for a review, see Stern, 2009). In healthy individuals, higher educational attainment (Arenaza-Urquijo *et al.*, 2013) as well as cognitive enrichment (Sun *et al.*, 2016) have been related to greater volume and greater metabolism in frontotemporal regions, thus likely enhancing brain performance (Barulli and Stern, 2013).

Genetic modifiers of disease expression also exist, affecting the phenotype and prognosis. *TMEM106B* has been identified as a genetic modifier in FTD, modulating the age at disease onset in frontotemporal lobar degeneration–TDP-43 disease (Cruchaga *et al.*, 2011; Gallagher *et al.*, 2014; van Blitterswijk *et al.*, 2014). The *TMEM106B* rs1990622 TT genotype is detrimental and associated with earlier age at disease onset (Cruchaga *et al.*, 2011) and greater functional impairment in frontal regions in presymptomatic *GRN* mutation carriers (Premi *et al.*, 2014). Conversely, the role of this polymorphism in *C9orf72* mutation carriers is still unclear, as it has been suggested a detrimental effect of *TMEM106B* rs1990622 CC genotype on disease onset and death (Deming and Cruchaga, 2014; Gallagher *et al.*, 2014; van Blitterswijk *et al.*, 2014).

In this study, we aimed to evaluate modifiers of structural brain changes in presymptomatic mutation carriers from a large international cohort of subjects at risk for genetic FTD, investigating the effect of (i) pathogenetic mutation, i.e. *MAPT*, *GRN* and *C9orf72* carriers versus non-carriers; (ii) cognitive reserve, as measured by years of formal schooling; and (iii) *TMEM106B* rs1990622 genotype, and their interaction, on grey matter volume.

We hypothesized that individually and together, these three factors will modulate the degree of structural atrophy.

## Materials and methods

### Participants

Data for this study were drawn from the GENFI multicentre cohort study (Rohrer *et al.*, 2015), which consists of 13 research centres in the UK, Italy, The Netherlands, Sweden, and Canada. Inclusion and exclusion criteria have been previously described (Rohrer *et al.*, 2015). Local ethics committees approved the study at each site and all participants provided written informed consent according to the Declaration of Helsinki. For the aim of the present work, we considered participants at 50% risk of carrying a *GRN*, *C9orf72* or *MAPT* mutation based on having a first-degree relative who was a known symptomatic mutation carrier. Between January 2012 and April 2015, 365 participants were recruited into GENFI, of which 294 were at risk and 71 symptomatic. Of the 294 at-risk participants, 22 did not have a T_1_-weighted MRI scan suitable for volumetric analysis. Included at-risk subjects underwent a careful recording of demographic data, including years of formal schooling (education), past medical history, and a standardized clinical and neuropsychological assessment, as previously published (Rohrer *et al.*, 2015). Genotyping was then performed for the *TMEM106B* rs1990622 (C/T) single nucleotide polymorphism according to standard procedures (Premi *et al.*, 2014) (at the individual sites in 70.6% of cases, and at the University of Brescia, Italy in the remaining 29.4%). Genotype was not available for 41 participants, and so the final analysis was performed on 231 participants: 108 presymptomatic mutation carriers [genetic status (GS) = 1], 61 with *GRN*, 33 with *C9orf72* and 14 with *MAPT* mutations) and 123 non-carriers (GS = 0)]. Participants (GS = 0 and GS = 1) came from 77 families (15 with *MAPT*, 33 with *GRN*, and 29 with *C9orf72* mutations). *TMEM106B* genotype distribution was comparable between groups (GS = 0 versus GS = 1, Pearson χ^2^ test, *P = *0.958), as well as among GS = 1 subgroups (i.e. *GRN*, *C9orf72* and *MAPT* mutation carriers, *P = *0.419). Demographic characteristics of GS = 1, subgrouped on the basis of mutation type, and GS = 0 are reported in Table 1. Mean age of GS = 1 was 45.9 years (range 20.5–70.5 years) and of GS = 0 was 48.3 years (range 19.4–85.7 years). No significant differences were found in age, gender, years of education, and neuropsychological tests between the groups.

**Table 1:** Demographic and clinical characteristics and *TMEM106B* genotype in the studied groups

	GS = 0 (*n = *123)	GS = 1 (*n = *108)	*C9orf72* (*n = *33)	*GRN* (*n = *61)	*MAPT* (*n = *14)	*P*-value
Age, years	48.3 ± 14.4	45.9 ± 11.3	43.6 ± 10.5	49.4 ± 10.6	36.4 ± 9.5	0.164^*^
Gender, % female (*n*)	63.4 (78)	64.8 (70)	57.6 (19)	65.6 (40)	78.6 (11)	0.891^†^
Education, years	13.7 ± 3.3	14.0 ± 3.1	13.8 ± 3.1	14.0 ± 3.2	14.3 ± 2.6	0.596^*^
**TMEM rs1990622**						0.958^†^
C/C (%)	11.4 (14)	10.1 (11)	9.1 (3)	9.8 (6)	14.3 (2)	
C/T (%)	51.2 (63)	51.9 (56)	51.5 (17)	57.4 (35)	28.6 (4)	
T/T (%)	37.4% (46)	38.0 (41)	39.4 (13)	32.8 (20)	57.1 (8)	
**Neuropsychological evaluation**						
CBI-Revised	3.28 ± 5.13	3.78 ± 7.00	5.14 ± 6.54	3.26 ± 7.70	3.07 ± 4.71	0.402^#^
MMSE	29.22 ± 1.25	29.14 ± 1.33	29.12 ± 1.32	29.00 ± 1.41	29.77 ± 0.83	0.211^#^
Logical Memory-Immediate Recall	0.54 ± 1.48	0.68 ± 1.45	0.76 ± 1.57	0.50 ± 1.35	1.25 ± 1.51	0.608^#^
Logical Memory-Delayed Recall	0.22 ± 1.10	0.33 ± 1.13	0.44 ± 1.23	0.19 ± 1.09	0.66 ± 1.03	0.597^#^
Digit Span forwards	0.11 ± 1.02	−0.02 ± 1.03	−0.12 ± 1.12	−0.03 ± 1.02	0.28 ± 0.84	0.225^#^
Digit Span backwards	−0.03 ± 0.95	−0.15 ± 0.92	−0.06 ± 1.01	−0.24 ± 0.86	0.05 ± 0.99	0.311^#^
Trail Making Test Part A	0.29 ± 0.75	0.28 ± 0.63	0.22 ± 0.62	0.22 ± 0.63	0.71 ± 0.52	0.352^#^
Trail Making Test Part B	0.29 ± 0.76	0.30 ± 0.83	0.21 ± 1.09	0.28 ± 0.66	0.62 ± 0.72	0.565^#^
Digit Symbol Task	0.34 ± 1.05	0.38 ± 1.00	0.34 ± 0.96	0.22 ± 0.96	1.14 ± 0.98	0.575^#^
Boston Naming Test	0.10 ± 0.88	0.11 ± 0.97	−0.13 ± 1.15	0.29 ± 0.68	−0.08 ± 1.41	0.933^#^
Letter Fluency	0.22 ± 1.02	0.21 ± 0.98	0.38 ± 0.94	0.09 ± 0.92	0.38 ± 1.27	0.593^#^
Category Fluency	−0.02 ± 1.07	−0.15 ± 1.32	−0.89 ± 1.12	0.23 ± 1.32	−0.02 ± 1.01	0.281^#^
Block Design	0.04 ± 0.99	0.15 ± 1.07	−0.24 ± 1.24	0.21 ± 0.94	0.78 ± 0.83	0.799^#^

GS = 0: mutation non-carriers; GS = 1: mutation carriers.

CBI = Cambridge Behavioural Inventory; MMSE = Mini-Mental State Examination.

Results are expressed as mean ± standard deviation or otherwise indicated. *P*-values, GS = 1 versus GS = 0 comparison: ^*^Student *t*-test; ^†^χ^2^ Chi-Square test; ^#^one-way ANCOVA (expressed as Z-scores).

### Imaging analysis

T_1_-weighted volumetric MRI scans were parcellated into cortical and subcortical regions as previously described (Rohrer *et al.*, 2015), using an atlas propagation and label fusion strategy (Cardoso *et al.*, 2015), combining regions of interest to calculate grey matter cortical volumes (separated into the frontal, temporal, parietal, occipital, cingulate, and insular cortices), subcortical volumes (hippocampus, amygdala, caudate, putamen, accumbens, pallidum, and thalamus), cerebellum volume (http://www.neuromorphometrics.org:808/seg/) (Diedrichsen *et al.*, 2009). Whole-brain volumes were measured using a semi-automated segmentation method (Freeborough *et al.*, 1997). All measures were expressed as a percentage of total intracranial volume (measured with SPM12 with a combination of grey matter, white matter, and CSF segmentations).

### Statistical analysis

**Figure 1:**
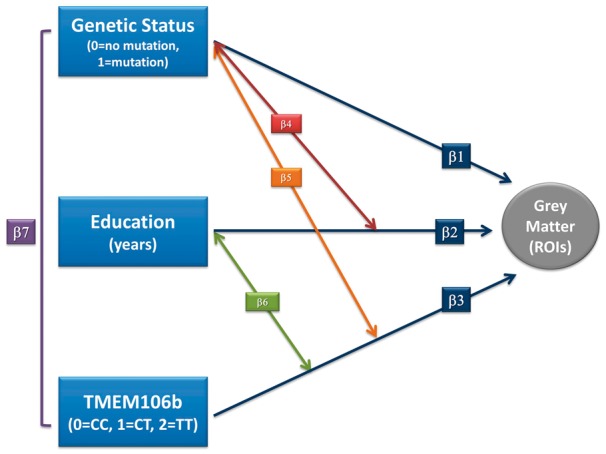
**Model design and results of interaction model on grey matter volume.** β1, β2, β3: main effect relationship of each factors (dark blue lines); β1: pathogenetic mutation (*GRN*, *MAPT* or *C9orf72*); β2: cognitive reserve as measured by years of formal education, and β3: *TMEM106B* rs1990622 polymorphism (coded as TT, TC and CC). β4, β5, β6: two-way interaction effect of each factor [β4: genetic status and education (red line), β5: genetic status and *TMEM106B* (orange line), and β6 (green line): education and *TMEM106B*]. β7: three-way interaction effect (genetic status, education, and *TMEM106B*) on grey matter volume (purple line). ROI = region of interest.

We fitted a linear mixed effect interaction model (Gałecki and Burzykowski, 2013). We assessed the main effect of three factors on grey matter: (i) the presence of pathogenetic mutation (GS, coded as GS = 1 for *GRN*, *MAPT or C9orf72* mutation carriers and GS = 0 for mutation non-carriers); (ii) the role of cognitive reserve as measured by years of formal education; and (iii) the *TMEM106B* rs1990622 C/T genotype (coded as CC, CT or TT). The relationship between each factor and grey matter volume was labelled as β1, β2, and β3, respectively (Fig. 1, dark blue lines). Furthermore, we considered the two-way interaction effect of each factor (i.e. GS and education, labelled as β4 (red line), GS and *TMEM106B* genotype, labelled as β5 (orange line), and education and *TMEM106B* genotype, labelled as β6 (green line) (Fig. 1). Finally, we considered the three-way interaction effect, i.e. GS, education, and *TMEM106B* genotype, on grey matter (β7) (Fig. 1). These main and interaction effects were adjusted by fixed covariates, namely age and gender. Moreover, we considered two random effect factors, study site and pedigree, which permitted analysis of the correlations of subjects in the same cluster (centres of subjects’ enrolment or individual families).

To overcome the complexity of multiple comparison corrections, we first carried out data reduction of grey matter parcellation data. We proposed a graph-Laplacian Principal Component Analysis (gLPCA) to obtain a low dimensional representation of grey matter parcellation, which incorporated graph structure (Jiang *et al.*, 2013). We did not apply principal component analysis (PCA), widely used to obtain a low-dimensional representation, as the imaging data presented spatial distribution and high left-right correlation (Belkin and Niyogi, 2001). Graph-Laplacian PCA (gLPCA) has several advantages: (i) it is modelled on the representation of the data; (ii) it can be easily calculated, presenting a compact closed-form solution; and (iii) it allows noise removal. Once we obtained data reduction, bivariate correlations between principal component (PC) scores and each grey matter measure were computed. Finally, we fitted the mixed-effect interaction models with grey matter, summarized by the first PC scores, as outcome variable. Statistical analysis was performed via R packages (www.r-project.org) and in-house R scripts.

## Results

By gLPCA using the skeleton graph between grey matter measures (Supplementary Fig. 1), the first PC (PC1) was selected to summarize the grey matter volume data. Frontal, parietal and temporal regions were the areas that contributed most to graph construction and PC1 scores, based on correlations between PC1 scores and grey matter measures (Supplementary Table 1).

Fitting the linear mixed-interaction model with fixed covariates (age and gender) and random effects (study site and pedigree), a significant direct effect of GS and years of education on grey matter outcome (PC1 scores) was observed (*P = *0.002 and *P = *0.02, respectively), while no effect of *TMEM106B* genotype on grey matter was detected. We did not find any significant two-way interaction between the considered variables, but did find a three-way interaction on grey matter (*P = *0.007) (Table 2).

**Table 2:** Output from the linear mixed effect interaction model

Fixed effects	β	Estimate	SE	Z-value	*P*-value
GS	**β 1**	**−0.262**	**0.084**	**−3.120**	**0.002**
Education	**β 2**	**0.035**	**0.015**	**2.350**	**0.020**
*TMEM106B*	β 3	−0.035	0.066	−0.522	0.600
GS^*^Education	β 4	−0.046	0.027	−1.729	0.080
GS × *TMEM106b*	β 5	0.055	0.133	0.413	0.680
Education × *TMEM106b*	β 6	−0.0005	0.020	−0.025	0.980
GS × Education × *TMEM106b*	**β 7**	**0.110**	**0.041**	**2.700**	**0.007**
Age	−	−0.052	0.003	−14.58	<0.001
Gender	−	−0.527	0.088	−6.008	<0.001
**Random effects**		**Variance**	**SE**		
Pedigree (no. groups = 77)	−	0.085	0.290		
Site (no. groups = 13)	−	0.004	0.063		

*TMEM106B* = *TMEM106B* rs1990622 polymorphism; SE = standard error; random effects = variance of the random intercept between groups. Bold values represent significant values of the studied effect (β1, β2, β7), as reported by P values column.

**Figure 2:**
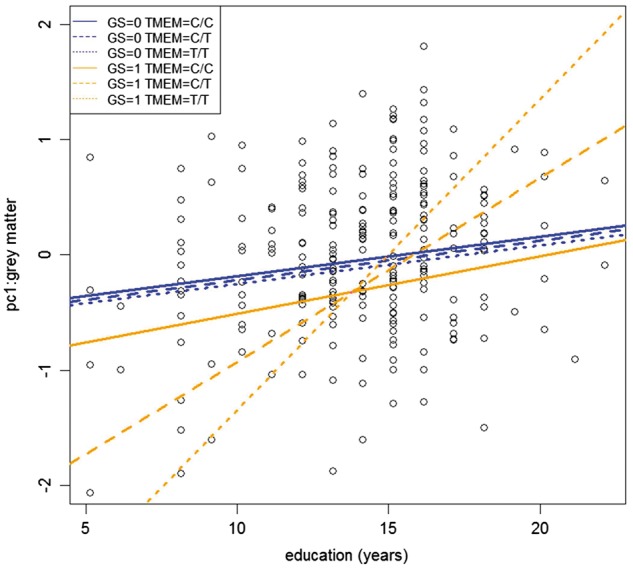
**Summary of the results from the fitted interaction model.***x*-axis, education attainment (years) *y*-axis, grey matter volume as obtained by considering principal component (PC) 1, GS = 1: mutation carriers; GS = 0: mutation non-carriers; TMEM = *TMEM106B.* See ‘Results’ section for details.

The data are summarized in Fig. 2. On the *x*-axis, years of education (i.e. cognitive reserve) are reported, and on the *y*-axis grey matter volume (PC1). Years of education had a significant direct effect on grey matter volume, independently of GS. We found that the greater the years of education, the greater the grey matter volume (in both GS = 0 and GS = 1), suggesting that cognitive reserve was able to exert an effect in presymptomatic at-risk participants carrying pathogenetic mutations as well as in non-carriers, by increasing grey matter volume.

In comparison to non-carriers (GS = 0, red line), mutation carriers (GS = 1, blue line) showed a significant decrease of grey matter volume, confirming the effect of pathogenetic mutations in shaping progressive atrophy before the onset of symptoms (Rohrer *et al.*, 2015).

The *TMEM106B* genotype did not exert a direct effect on grey matter volume, and it did not affect grey matter (Fig. 2, red line, GS = 0 and *TMEM106B* CC or CT or TT). However, in those individuals carrying pathogenetic mutations (GS = 1), *TMEM106B* polymorphism modulated the slope of the relationship between education and grey matter volume (GS^*^Education^*^TMEM106B): a steeper slope was found in *TMEM106B* TT carriers compared with CT carriers, which in turn was greater than that of CC carriers, with a dose-dependent effect (Fig. 2, green and purple lines). Considering the contribution of each mutation separately, the effect of *TMEM106B* genotype (GS^*^Education^*^TMEM106B) was mainly driven by *C9orf72* mutation carriers, the subgroup of patients with the greatest atrophy (Supplementary Table 2 and Supplementary Fig. 2).

## Discussion

Autosomal dominant FTD presents with significant inter- and intra-familial variability among individuals bearing the same pathogenetic mutation. This suggests the presence of environmental, genetic and/or epigenetic modifiers, influencing the age at disease onset and clinical phenotype (Borroni and Padovani, 2013). The effect of genetic modifiers and environmental factors that might trigger the onset of neurodegeneration in carriers of the pathogenetic mutation (which is present at birth but only manifests symptoms in mid-late adulthood) are of extreme interest. In this view, the pathogenesis of inherited FTD may be a model of ‘Latent Early-Life Associated Regulation’ (LEARn), in which latent expression of associated genes is triggered by environmental and non-environmental factors (Maloney *et al.*, 2012; Maloney and Lahiri, 2016), with neurodegeneration being modulated by lifetime exposure to one or more environmental factors as well as genetic background.

In the present study, we aimed at identifying modulating factors of neuronal loss in presymptomatic subjects bearing pathogenetic mutations within *GRN*, *MAPT* and *C9orf72* genes through the surrogate marker of volumetric MRI.

We analysed the effect of (i) pathogenetic mutations; (ii) cognitive reserve as measured by years of schooling; and (iii) *TMEM106B* genotype on grey matter volume in the large GENFI cohort. Indeed, we chose grey matter volume as an endpoint measure as it correlates well with indexes of disease severity (Premi *et al.*, 2016) and progression (Brambati *et al.*, 2007; Lam *et al.*, 2014).

First, we confirmed, as previously reported (Rohrer *et al.*, 2015), that pathogenetic mutations are detrimental to grey matter volume years before expected age at disease onset, being associated with smaller volumes i.e. greater atrophy as compared to siblings who did not inherit the mutation. *C9orf72* repeat expansion carriers had a greater degree of atrophy (Rohrer *et al.*, 2015), as compared to *GRN* mutation carriers and *MAPT* mutation carriers, with the latter being the smallest group in our sample.

Furthermore, we demonstrated that cognitive reserve is associated with brain atrophy and also modulate neuronal loss years before the onset of symptoms. *TMEM106B* polymorphism, on the other hand, only modulated grey matter volume in those with an autosomal dominant mutation and with the lowest education.

The duration of formal schooling, as a proxy of cognitive reserve, was associated with greater grey matter volume in both non-carriers and in mutation carriers. This might suggest that those subjects with higher educational attainment were able to better counteract the detrimental effect of a pathogenetic mutation than their counterparts with lower education. However, this effect was also found in the group that did not carry mutations: those with higher education attainment had greater grey matter volume then those with low education. Hence the finding is not specific to mutation carriers suggesting a broader effect of cognitive reserve on maintaining and ameliorating brain functioning. Interestingly, even if presymptomatic mutation carriers already had mild structural changes (Rohrer *et al.*, 2015), the relationship between years of education and structural changes was comparable to that observed in non-carriers (direct correlation, the higher the education the greater the grey matter volume) (Sole-Padulles *et al.*, 2009; Rzezak *et al.*, 2015), rather than that reported in symptomatic FTD (inverse correlation, the higher the education the lower grey matter volume) (Borroni *et al.*, 2009). The concept of cognitive reserve was originally proposed to explain the lack of a direct relationship between the degree of brain pathology and the severity of the clinical manifestations that should supposedly result from such damage in neurodegenerative disorders such as Alzheimer’s disease (Stern *et al.*, 1992, 1994) and FTD (Borroni *et al.*, 2009; Premi *et al.*, 2012, 2013). Cognitive reserve represents the hypothesized capacity of the adult brain to compensate for the effects of a disease or injury that would be sufficient to cause clinical dementia in an individual with less cognitive reserve (Stern, 2002). Herein, we propose that high education might postpone the onset of dementia in those subjects at risk of developing FTD (Akbaraly *et al.*, 2009; Craik *et al.*, 2010; Pettigrew *et al.*, 2013). These findings extend previous results obtained in healthy subjects (Sole-Padulles *et al.*, 2009; Rzezak *et al.*, 2015), and might represent a possible strategy to delay onset of inherited FTD.

Conversely to education attainment, *TMEM106B* genotype did not have any effect on mutation free individuals, but this genetic trait might represent an additional non-modifiable risk factor in mutation carriers. Literature data have widely proven that *TMEM106B* variants are genetically associated to frontotemporal lobar degeneration–TDP-43 pathology and are considered a major risk factor for this disease (Chen-Plotkin *et al.*, 2012; Busch *et al.*, 2013; Nicholson and Rademakers, 2016). It has been suggested that the *TMEM106B* polymorphism might modulate progranulin plasma levels, thus affecting age at onset of symptoms in *GRN* mutation carriers and explaining in part the reported variability (Cruchaga *et al.*, 2011). Furthermore, presymptomatic *GRN* mutation carriers bearing the *TMEM106B* TT genotype showed greater functional brain damage than those with CT/CC *TMEM106B* genotypes (Premi *et al.*, 2014). In frontotemporal lobar degeneration–TDP-43 due to *C9orf72* mutations, the relationship is less clear, and it has been suggested that *TMEM106B* might be able to affect disease pathology, but with an opposite association (Gallagher *et al.*, 2014; van Blitterswijk *et al.*, 2014): two independent groups analysed the association of *TMEM106B* variants with disease risk, age at onset, and age at death in *C9orf72* expansion carriers with the CC genotype (protective in *GRN* carriers) found to be associated with earlier onset and earlier death in *C9orf72* expansion carriers (Deming and Cruchaga, 2014; Gallagher *et al.*, 2014; van Blitterswijk *et al.*, 2014). This effect may be an example of the general phenomenon of epistasis, in which a genetic variant is beneficial on some genetic backgrounds but deleterious in others (Gallagher *et al.*, 2014; Busch *et al.*, 2016). In particular, as hypothesized, if in *GRN*-related TDP-43 pathology *TMEM106B* is related to endosomal-lysosomal dysfunction and to the perturbation of the progranulin pathway, in *C9orf72* knockdown mice *TMEM106B* over-expression may produce a phenotypic rescue effect (Busch *et al.*, 2016). However, further studies are needed to elucidate its mechanism of action. Another possibility is that *TMEM106B* is simply in linkage disequilibrium with the actual associated variant and when different populations are examined, the allele associated with disease modulation is different.

In the present work, a moderating, dose dependent effect of *TMEM106B* rs1990622 genotype together with education attainment was observed on grey matter volume in presymptomatic subjects carrying pathogenetic mutations. This finding supports the idea that epigenetic modifications in *TMEM106B* might occur. Epigenetic mechanisms, mostly mediated by DNA methylation, have been shown to be important in other neurodegenerative disorders (Piaceri *et al.*, 2015) and to be influenced by socioeconomic status, which is strongly associated with cognitive reserve (Tehranifar *et al.*, 2013). Thus, it could be hypothesized that *TMEM106B*, a gene containing a number of methylation sites (http://genome.ucsc.edu), might exert its effect on structural changes in at-risk subjects via cognitive reserve. Future studies, however, need to be performed to confirm this hypothesis.

We indeed found a detrimental effect of the CC genotype, but the present results were mainly driven by subjects carrying *C9orf72* mutations, in which CC is the risk genotype (Supplementary Table 2).

We acknowledge that there are limitations with this work. First, the correlation between the factors herein considered and age at disease onset would benefit from longitudinal follow-up and independent studies to confirm the present results. The lack of assessment of leisure activities prevents us from characterizing the entire spectrum of cognitive reserve proxies (Nucci *et al.*, 2012), and we are aware of possible biases determined by different school systems across the involved countries. Finally, the role of *TMEM106b* genotype should be further evaluated in the different genetic groups alone.

In conclusion, our findings indicate that even several years before the onset of symptoms, brain changes in inherited FTD may be modulated by environmental and genetic factors. In the absence of effective pharmacotherapeutic treatments for counteracting the onset of symptoms in pathogenetic mutation carriers, high education may represent a large-scale strategy to be considered by national health system policies.

*TMEM106b* genotype needs to be considered as an extra non-modifiable trait affecting brain pathology, and each FTD mutation should be analysed individually. Future clinical trials in genetic FTD should take into account both education level and *TMEM106b* genotype to define subjects with greater brain damage, thus representing those at higher risk of developing FTD at an earlier age.

## Funding

This work was supported in part by operating grants from the Canadian Institutes of Health Research (CIHR) to M.M. (Centres of Excellence in Neurodegeneration/CIHR: INE117893 and CIHR: MOP137116) and from Italian Ministry of Health to F.T.

## Supplementary material

Supplementary material is available at *Brain* online.

## Supplementary Material

Supplementary Data

## Abbreviations

FTD: frontotemporal dementia
GS: genetic status

## Appendix 1

### GENFI consortium members

See Supplementary material for consortium member affiliations.

Christin Andersson, Andrea Arighi, Luisa Benussi, Giuliano Binetti, Sandra Black, Katrina Dick, Marie Fallström, Carlos Ferreira, Chiara Fenoglio, Nick Fox, Morris Freedman, Giorgio Fumagalli, Roberta Ghidoni, Marina Grisoli, Vesna Jelic, Lize Jiskoot, Ron Keren, Gemma Lombardi, Carolina Maruta, Lieke Meeter, Gabriel Miltenberger-Miltényi, Benedetta Nacmias, Linn Öijerstedt, Jessica Panman, Michela Pievani, Cristina Polito, Sara Prioni, Rosa Rademakers, Veronica Redaelli, Ekaterina Rogaeva, Giacomina Rossi, Martin Rossor, Elio Scarpini, David Tang-Wai, David Thomas, Håkan Thonberg, Pietro Tiraboschi, Rick van Minkelen, Ana Verdelho, Jason Warren.
