# Supplementary material for: Cognitive reserve and TMEM106B genotype modulate brain damage in presymptomatic frontotemporal dementia: a GENFI study
Source: Brain. 2017 Apr 27;140(6):1784–91. doi: 10.1093/brain/awx103 (PMC5445253; doi:10.1093/brain/awx103)
Supplement: Supplementary Data [file awx103_Supp.zip › awx103-suppl_data/brain-2016-02105-File005.pdf]

Supplementary Table 1. Principal component coefficients and correlations of the first Principal Component (PC1) with each Region of Interest (ROI) extracted from gLPCA

ROI	coefficients	correlations
**Frontal lobe, L**	-0.503	**-0.911**
**Frontal lobe, R**	-0.509	**-0.902**
**Parietal lobe, L**	-0.266	**-0.806**
**Parietal lobe, R**	-0.262	**-0.802**
**Temporal lobe, L**	-0.222	**-0.707**
**Temporal lobe, R**	-0.238	**-0.707**
**Cerebellum**	-0.445	**-0.633**
**Thalamus, L**	-0.029	**-0.571**
**Thalamus, R**	-0.030	**-0.559**
**Insula, L**	-0.024	**-0.553**
**Insula, R**	-0.027	**-0.548**
**Hippocampus, R**	-0.020	**-0.538**
**Hippocampus, L**	-0.018	**-0.515**
**Cingulate, L**	-0.044	**-0.508**
**Occipital lobe, R**	-0.135	-0.495
**Occipital lobe, L**	-0.136	-0.483
**Putamen, R**	-0.017	-0.426
**Putamen, L**	-0.016	-0.412
**Cingulate, R**	-0.037	-0.385
**Accumbens, L**	-0.003	-0.380
**Accumbens, R**	-0.003	-0.374
**Pallidum, L**	-0.002	-0.290
**Amygdala, R**	-0.002	-0.262
**Amygdala, L**	-0.002	-0.260
**Caudate, R**	-0.007	-0.184
**Pallidum, R**	-0.001	-0.165
**Caudate, L**	-0.006	-0.162

gLPCA: Graph-Laplacian Principal Component Analysis;

ROI: region of interest; L: left; R: right. In bold face the highest correlations (>0.5).

Supplementary Table 2. P-values from the linear mixed effect interaction model.

	β	GS	GRN	MAPT	C9orf72
Carriers : non carriers		**108 : 123**	**61 : 170**	**14 : 217**	**33 : 198**
GS	β1	**0.002**	0.385	0.239	**0.000**
Education	β2	**0.020**	**0.047**	**0.016**	**0.019**
TMEM106b	β3	0.600	0.567	0.487	0.886
GS*Education	β4	0.080	0.105	**0.027**	0.485
GS**TMEM106b*	β5	0.680	0.077	0.305	**0.043**
Education**TMEM106b*	β6	0.980	0.888	0.961	0.637
GS*Education**TMEM106b*	β7	**0.007**	0.227	0.165	**0.026**

Principal Component 1 (PC1) score was the outcome variable and Genetic Score (GS) was computed considering all the pathogenetic mutations, i.e. *MAPT or GRN or C9orf72* carriers, and each mutation separately. GS= genetic score; *MAPT: Microtubuli Associated Protein Tau; GRN: Granulin; C9orf72: chromosome 9 open reading frame 72.*

Legend to Supplementary Figure 1. The skeleton graph resulting from grey matter (GM) volume measurers from the PC algorithm (http://www.jstatsoft.org/v47/i11) on the GM correlation matrix, setting the significance level for the individual conditional independence tests alpha=0.10.

Legend to Supplementary Figure 2. Regression lines of the results from the fitted three-way interaction model in different genetic subgroups.

x-axis, education attainment (years)**;** y-axis, grey matter volume as obtained by considering Principal Component 1.

g=genetic score; g=1, mutation carriers; g=0, mutation non-carriers; GS: all sample; GRN: *Granulin*, MAPT: *microtubule-associated protein tau, C9orf72*: *chromosome 9 open reading frame 72.*

TMEM=*TMEM106B;* (carriers: non carriers) between brackets

See result section for further details.
