# Supplementary material for: Cognitive reserve and TMEM106B genotype modulate brain damage in presymptomatic frontotemporal dementia: a GENFI study
Source: Brain. 2017 Apr 27;140(6):1784–91. doi: 10.1093/brain/awx103 (PMC5445253; doi:10.1093/brain/awx103)
Supplement: Supplementary Data [file awx103_Supp.zip › awx103-suppl_data/brain-2016-02105-File009.pdf]

Christin Andersson[27], Andrea Arighi[4], Luisa Benussi[13], Giuliano Binetti[13], Sandra Black[6], Katrina Dick[20], Marie Fallström[26], Carlos Ferreira[29], Chiara Fenoglio[4], Nick Fox[20], Morris Freedman[22], Giorgio Fumagalli[4], Roberta Ghidoni[13], Marina Grisoli[8], Vesna Jelic[5,26], Lize Jiskoot[3], Ron Keren[23], Gemma Lombardi[16], Carolina Maruta[15], Lieke Meeter[3], Gabriel Miltenberger-Miltényi[15], Benedetta Nacmias[16], Linn Öijerstedt[5,27], Jessica Panman[3], Michela Pievani[13], Cristina Polito[25], Sara Prioni[9], Rosa Rademakers[31], Veronica Redaelli[9], Ekaterina Rogaeva[8], Giacomina Rossi[9], Martin Rossor[20], Elio Scarpini[4], David Tang-Wai[23], David Thomas[20], Håkan Thonberg[5,28], Pietro Tiraboschi[8], Rick van Minkelen[24], Ana Verdelho[30], Jason Warren[20]

[1] Neurology Unit, Department of Clinical and Experimental Sciences, University of Brescia, Brescia, Italy

[2] Department of Brain and Behavioral Science, Medical and Genomic Statistics Unit, University of Pavia, Italy

[3] Department of Neurology, Erasmus Medical Center, Rotterdam, Netherlands

[4] Dept. of Pathophysiology and Transplantation, "Dino Ferrari" Center, University of Milan, Fondazione Cà Granda, IRCCS Ospedale Maggiore Policlinico, Milan, Italy

[5] Karolinska Institutet, Department NVS, Center for Alzheimer Research, Division of Neurogenetics, Sweden

[6] Department of Geriatric Medicine, Karolinska University Hospital-Huddinge, Stockholm, Sweden

[7] LC Campbell Cognitive Neurology Research Unit, Department of Medicine, Division of Neurology, Sunnybrook Health Sciences Centre, Sunnybrook Research Institute, University of Toronto, Toronto, ON, Canada

[8] Toronto Western Hospital, Tanz Centre for Research in Neurodegenerative Disease, Toronto, ON, Canada

[9] Fondazione Istituto di Ricovero e Cura a Carattere Scientifico Istituto Neurologico Carlo Besta, Milano, Italy

[10] Department of Clinical Neurosciences, University of Cambridge, Cambridge, UK

[11] Clinique Interdisciplinaire de Mémoire, Département des Sciences Neurologiques, CHU de Québec, and Faculté de Médecine, Université Laval, QC, Canada

[12] Department of Clinical Neurological Sciences, University of Western Ontario, London, ON, Canada

[13] Istituto di Ricovero e Cura a Carattere Scientifico Istituto Centro San Giovanni di Dio Fatebenefratelli, Brescia, Italy

[14] Memory Clinic and LANVIE-Laboratory of Neuroimaging of Aging, University Hospitals and University of Geneva, Geneva, Switzerland

[15] Faculty of Medicine, University of Lisbon, Lisbon, Portugal

[16] Department of Neuroscience, Psychology, Drug Research and Child Health, University of Florence, Florence, Italy

[17] Istituto di Ricovero e Cura a Carattere Scientifico (IRCCS) “Don Gnocchi”, Florence, Italy

[18] Department of Laboratories, III Laboratory of Analysis, Brescia Hospital, Brescia, Italy

[19] Neuroradiology Unit, University of Brescia, Italy

[20] Dementia Research Centre, Department of Neurodegenerative Disease, UCL Institute of Neurology

[21] Centre for Medical Image Computing, UCL, UK

[22] Division of Neurology, Mount Sinai Hospital, University of Toronto, ON, Canada

[23] University Health Network Memory Clinic, Toronto Western Hospital, Toronto, ON, Canada

[24] Department of Clinical Genetics, Erasmus Medical Center, Rotterdam, The Netherlands

[25] Department of Clinical Pathophysiology, Nuclear Medicine Division, University of Florence, Florence, Italy

[26] Department of Geriatric Medicine, Karolinska University Hospital-Huddinge, Stockholm, Sweden

[27] Department of Clinical Neuroscience, Karolinska Institutet, Stockholm, Sweden

[28] Center for Alzheimer Research, Division of Neurogeriatrics, Karolinska Institutet, Stockholm, Sweden

[29] Instituto Ciências Nucleares Aplicadas à Saúde, Universidade de Coimbra, Coimbra, Portugal

[30] Department of Neurosciences, Santa Maria Hospital, University of Lisbon, Portugal

[31] Department of Neurosciences, Mayo Clinic, Jacksonville, Florida, USA
