# Supplementary figures and images for: Cognitive reserve and TMEM106B genotype modulate brain damage in presymptomatic frontotemporal dementia: a GENFI study
Source: Brain. 2017 Apr 27;140(6):1784–91. doi: 10.1093/brain/awx103 (PMC5445253; doi:10.1093/brain/awx103)

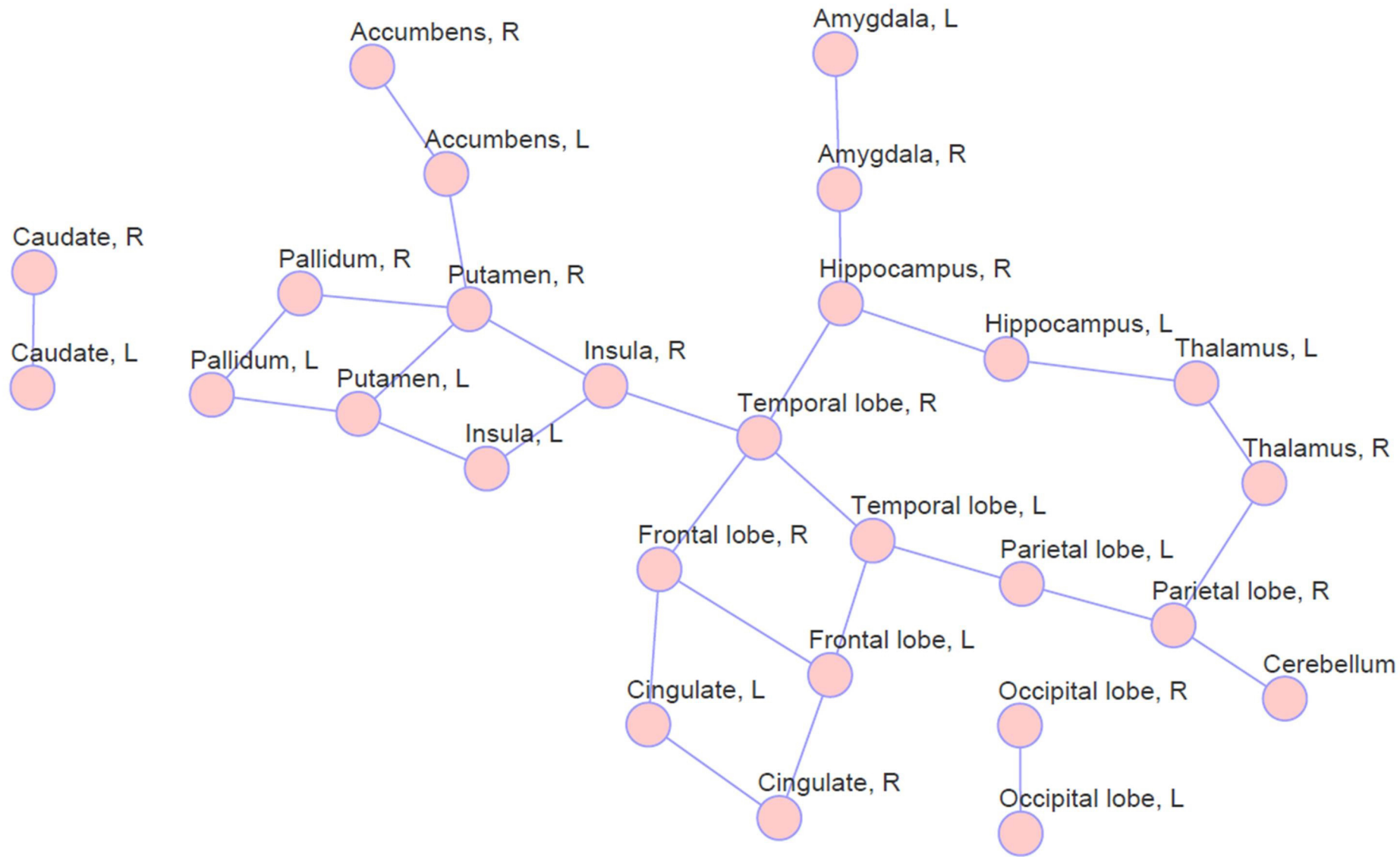

Supplement: Supplementary Data [file awx103_Supp.zip › awx103-suppl_data/brain-2016-02105-File006.pdf]

**g=GS(108:123)**

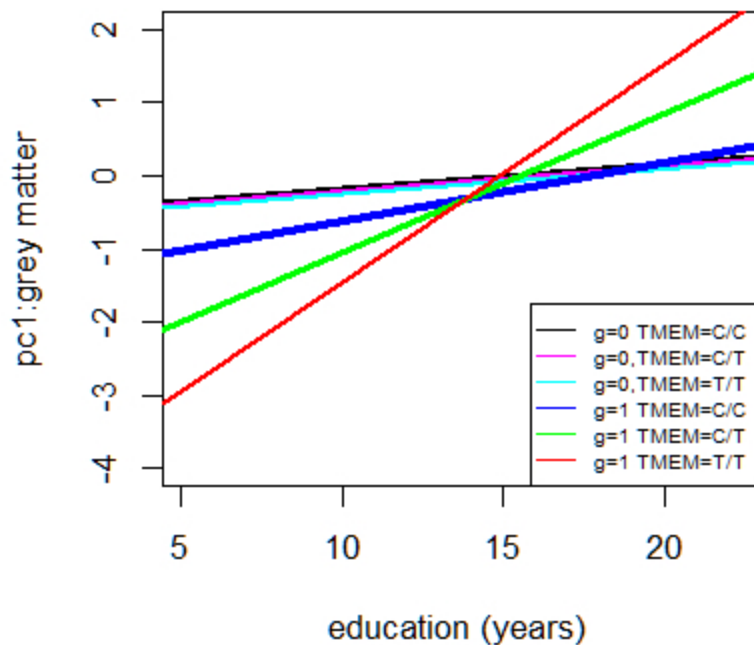

**g=GRN(61:170)**

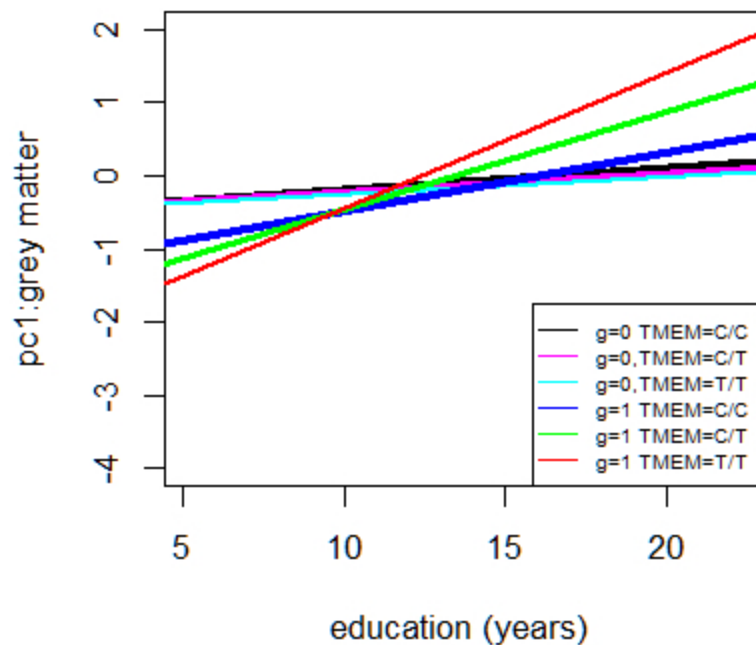

**g=MAPT(14:127)**

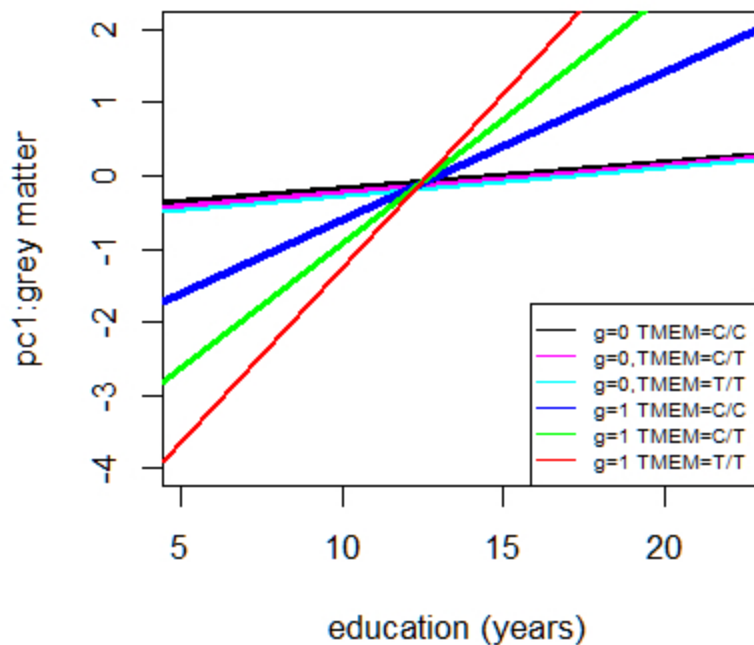

**g=C9orf72(33:198)**

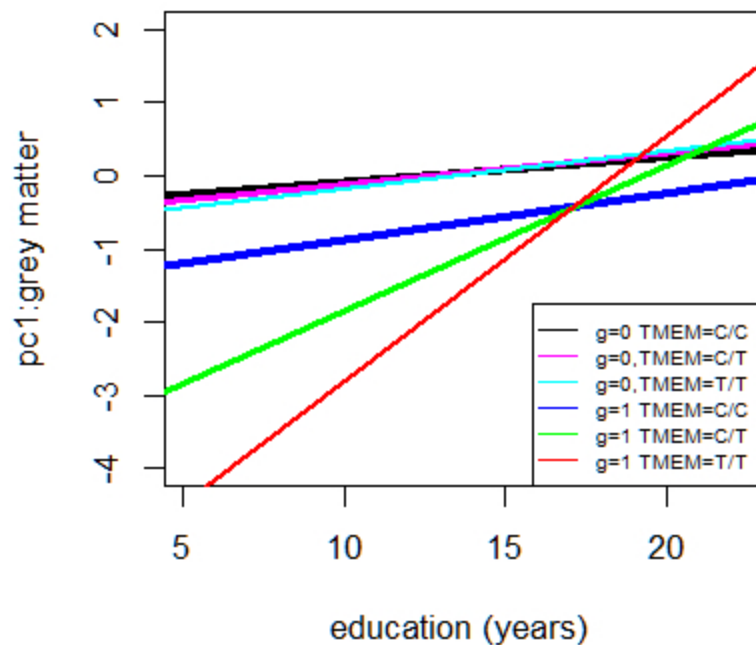

Supplement: Supplementary Data [file awx103_Supp.zip › awx103-suppl_data/brain-2016-02105-File007.pdf]
